# Influence of Old Age on Risk of Lymph Node Metastasis and Survival in Patients With T1 Colorectal Cancer: A Population-Based Analysis

**DOI:** 10.3389/fonc.2021.706488

**Published:** 2021-10-13

**Authors:** Hua Ye, Bin Zheng, Qi Zheng, Ping Chen

**Affiliations:** ^1^ Department of Gastrointestinal and Hernia Ward, HwaMei Hospital, University of Chinese Academy of Sciences, Ningbo, China; ^2^ Ningbo Institute of Life and Health Industry, University of Chinese Academy of Sciences, Ningbo, China; ^3^ Key Laboratory of Diagnosis and Treatment of Digestive System Tumors of Zhejiang Province, Ningbo, China; ^4^ Department of Endoscopy Center, YinZhou JiangShan MaoShan Hospital, Ningbo, China

**Keywords:** colorectal cancer, lymph node metastasis, elderly patients, survival, SEER

## Abstract

**Background:**

We aimed at determining the influence of old age on lymph node metastasis (LNM) and prognosis in T1 colorectal cancer (CRC).

**Methods:**

We collected data from eligible patients in Surveillance, Epidemiology, and End Results database between 2004 and 2015. Independent predictors of LNM were identified by logistic regression analysis. Cox regression analysis, propensity score-matched analysis, and competing risks analysis were used to analyze the associations between old age and lymph node (LN) status and to validate the prognostic value of old age on cancer-specific survival (CSS).

**Results:**

In total, 10,092 patients were identified. Among them, 6,423 patients (63.6%) had greater than or equal to 12 examined lymph nodes (LNE ≥12), and 5,777 patients (57.7%) were 65 years or older. The observed rate of LNM was 4.6% (15 out of 325) in T1 CRC elderly patients, with tumor size <3 cm, well differentiated, with negative carcinoembryonic antigen (CEA) level, and adenocarcinoma. Logistic regression models demonstrated that tumor size ≥3 cm (odds ratio, OR = 1.316, *P* = 0.038), poorly differentiated (OR = 3.716, *P* < 0.001), older age (OR = 0.633 for ages 65–79 years, OR = 0.477 for age over 80 years, both *P <*0.001), and negative CEA level (OR = 0.71, P = 0.007) were independent prognostic factors. Cox regression analysis demonstrated that CSS was not significantly different between elderly patients undergoing radical resection with LNE ≥12 and those with LNE <12 (hazard ratio = 0.865, *P* = 0.153), which was firmly validated after a propensity score-matched analysis by a competing risks model.

**Conclusions:**

The predictive value of tumor size, grading, primary site, histology, CEA level, and age for LNM should be considered in medical decision making about local resection. We found that tumor size was <3 cm, well differentiated, negative CEA level, and adenocarcinoma in elderly patients with T1 colorectal cancer which was suitable for local excision.

## Introduction

Colorectal cancer (CRC) is among the most prevalent malignant tumors in most countries worldwide and ranks third in cancer-associated deaths in the United States (1, 2). In addition, the incidence of CRC is rising rapidly, greatly threatening the health of the elderly. T1 CRC is defined as tumor invasion into the submucosa (through the muscularis mucosa but not penetrating into the muscularis propria). The tumor node metastasis stage system reveals an extremely similar survival for colon and rectal carcinoma, which, therefore, share the same staging system (3). Lymph node metastasis (LNM) has been uncovered to range from 8 to 15% in T1 CRC (4). Due to the substantial prognostic impacts of lymph node (LN) status, whether LN is involved is taken into consideration in clinical practice. To be specific, more comprehensive assessment of LN status is more likely to attenuate the risk of tumor understaging, while node-positive patients might be inaccurately identified as node-negative patients by insufficient evaluation, further leading to improper therapeutic approaches. Therefore, according to previous findings, most guidelines and consensus have recommended the assessment of 12 or more LNs for acceptable staging of CRC (3). However, the understaging mechanism has been argued in recent researches, which indicated limited improvement on survival by enhancing the number of sampled LNs by the efforts of professional associations as well as payers. Moreover, enhancing the number of sampled LNs during operation could not improve the survival of CRC patients 65 years and older (5). For one thing, overtreatment in patients could cause harmful responses (including unnecessary biopsy, surgical resection, and other therapeutic interventions), particularly in the elderly patients. For another thing, the incomplete removal of positive LNs could enhance the risk of a local recurrence, thereby leading to poor prognosis.

Advanced endoscopic techniques have been accepted as proper therapeutic interventions in T1 CRC patients following cautious selection and assessment (4, 6, 7). Local excision in T1 CRC patients could decrease the morbidity and further enhance the quality of life. In addition, careful local excision and a cautious assessment of excluding risk factors (including LNM) could avoid an unnecessary additional surgical intervention. Thus, patients at high risks of LNM should be identified, especially in the elderly, to establish appropriate therapeutic strategies and, simultaneously, to minimize the local relapse rate. Elderly CRC patients have a higher risk of death from non-tumor events than the overall population, including underlying diseases, infections, and cerebrovascular and cardiovascular accidents, thereby decreasing cancer-related mortality rate. Unfortunately, these is the issue which frequently occurs when making a prognostic prediction for elderly patients as the old population possesses a high frequency of frailty and comorbidities, exhibiting increased mortality from other causes, among those with cancer. At this time, the use of competing risks concept can be a good solution to this problem. In the case of competing risks, a single univariate analysis can be carried out by calculating the cumulative incidence function (CIF) of concern events and competitive events. Thus, the precise prognostic prediction has become more difficult, and it is urgent to establish reliable and discriminative approaches for prognostic prediction in elderly patients. Therefore, to minimize the local recurrence rate, patients with a high risk of LNM have to be identified.

To this end, with the logistic regression model, propensity score-matching (PSM) analysis, and competing risks approach, in this study, we explored the predictors for LNM and survival of elderly patients in T1 CRC by extracting eligible data from the Surveillance, Epidemiology, and End Results (SEER) database.

## Materials and Methods

### Study Population

The National Cancer Institute-supported SEER database records data on tumor incidence and survival by covering almost 28% of the population in the USA from diverse geographic regions (18 cancer registries) from 2004 to 2015. The collection and recoding of SEER data were performed using data items and codes on the basis of the North American Association of Central Cancer Registries (8). Access to the SEER database was obtained, and our study gained institutional approval. The clinicopathological characteristics of the selected patients are shown in **Table 1**.

**Table 1:** Clinicopathological characteristics of the selected patients.

Characteristic	Examined lymph nodes (LNE) <12	LNE ≥12	Statistic	*p*
	*N* = 3,669, %	*N* = 6,423, %		
Gender			*χ* ^2^ = 11.419	0.001
Female	1,694 (46.2)	3,190 (49.7)		
Male	1,975 (53.8)	3,233 (50.3)		
Age (years)			*Z* = -6.823	<0.001
Up to 49	209 (5.7)	563 (8.8)		
50–64	1,229 (33.5)	2,314 (36.0)		
65–79	1,589 (43.3)	2,648 (41.2)		
80+	642 (17.5)	898 (14.0)		
Race			*χ* ^2^ = 2.452	0.293
White	2,893 (78.8)	5,122 (79.7)		
Black	422 (11.5)	674 (10.5)		
Others^a^	354 (9.6)	627 (9.8)		
Lymph node metastasis			*χ* ^2^ = 17.38	<0.001
No	3,230 (88.0)	5,463 (85.1)		
Yes	439 (12.0)	960 (14.9)		
Tumor size (cm)			*Z* = -2.463	0.014
<1	489 (13.3)	848 (13.2)		
1–1.9	1,094 (29.8)	1,857 (28.9)		
2–2.9	749 (20.4)	1,545 (24.1)		
3+	611 (16.7)	1,338 (20.8)		
Not stated	726 (19.8)	835 (13.0)		
Year of diagnosis			*Z* = -30.354	<0.001
2004–2006	1,675 (45.7)	1,230 (19.1)		
2007–2009	916 (25.0)	1,582 (24.6)		
2010–2012	638 (17.4)	1,747 (27.2)		
2013–2015	440 (12.0)	1,864 (29.0)		
Marital status			*χ* ^2^ = 11.946	0.003
Married	2,152 (58.7)	3,817 (59.4)		
Single/widowed	1,017 (27.7)	1,611 (25.1)		
Other/unknown	500 (13.6)	995 (15.5)		
Grade			*χ* ^2^ = 18.837	0.001
Well-differentiated	653 (17.8)	1,062 (16.5)		
Moderately differentiated	2,485 (67.7)	4,342 (67.6)		
Poorly differentiated	251 (6.8)	563 (8.8)		
Undifferentiated	24 (0.7)	64 (1.0)		
Unknown	256 (7.0)	392 (6.1)		
Primary site			*χ* ^2^ = 367.941	<0.001
Cecum	389 (10.6)	1,049 (16.3)		
Ascending colon	387 (10.5)	1,385 (21.6)		
Hepatic flexure	88 (2.4)	239 (3.7)		
Transverse colon	303 (8.3)	472 (7.3)		
Splenic flexure	73 (2.0)	125 (1.9)		
Descending colon	211 (5.8)	237 (3.7)		
Sigmoid colon	1,232 (33.6)	1,486 (23.1)		
Rectum/rectosigmoid junction	986 (26.9)	1,430 (22.3)		
CEA			*χ* ^2^ = 46.226	<0.001
Positive	267 (7.3)	487 (7.6)		
Negative	1,341 (36.5)	2,769 (43.1)		
Borderline/unknown	2,061 (56.2)	3,167 (49.3)		
Histology			*χ* ^2^ = 0.974	0.615
Adenocarcinoma	3,446 (93.9)	6,014 (93.6)		
Mucinous carcinoma	204 (5.6)	366 (5.7)		
Signet ring cell carcinoma	19 (0.5)	43 (0.7)		

a American Indian/Alaska native, Asian/Pacific islander.

### Assessments and Data Acquisition

The SEER*Stat software was developed by the National Cancer Institute (Surveillance Research Program, National Cancer Institute SEER*Stat software 8.3.6; https://seer.cancer.gov). We conducted a comprehensive analysis of all primary CRC cases registered in the SEER database of the United States National Cancer Institute from 2004 to 2015. Patients were enrolled if (1) they were 18 years or older, (2) at least one LN was sampled, (3) they underwent surgery of T1 CRC, (4) the histological type included adenocarcinoma (8140), mucinous adenocarcinoma (MAC) (8480), and signet ring cell cancer (SRCC) (8490), and (5) they were actively followed up. The patients were eliminated if (1) they had distant metastasis, (2) they received adjuvant radiotherapy, (3) they had more than one type of malignancies, except those with CRC as the first diagnosed, (4) they had survival of less than 1 month, which was mostly caused by surgical complications, and (5) they only had a death certificate or were unaware whether an operation was conducted.

### Statistical Analysis

Data on age at diagnosis, race, year of diagnosis, marital status, gender, tumor size, tumor site, differentiation grade, survival (months), number of examined LNs, LNM, carcinoembryonic antigen (CEA) level, and death cause were collected from SEER database.

Overall survival (OS) as well as cancer-specific survival (CSS) were taken as outcomes according to specific codes. Non-oncological death was considered as a competitive event. In order to identify the prognostic factors with significant correlation with CSS, there would be overestimation of the cumulative incidence of every variable if the conventional Kaplan–Meier method was employed (9). In this condition, we should calculate the CIF instead of the KM method in univariate analyses. To be specific, CIF can calculate the incidence of interest endpoint events and competitive risk events, which accurately show the incidence of interest endpoint events after correction of competitive risk events (10).

Continuous data were compared using one-way ANOVA, and categorical data were compared by Pearson’s chi-square test or Fisher’s exact test. Both univariate and multivariate logistic regression models were adopted to explore and validate the risk factors for LNM [shown with odd ratios (ORs) along with 95% confidence intervals (CIs)]. Afterwards, both univariate and multivariate Cox regression analyses were employed to calculate the adjusted hazard ratios (HRs) and 95% CIs. Additionally, a PSM was performed by a 1:2 “nearest neighbor” match paradigm for adjustment of different general information and for bias minimization. Histology, age, marital status, year of diagnosis, LNM, gender, CEA level tumor size as well as primary tumor site were used as covariates. After matching, we subsequently compared two groups with control for covariate balance and similarity in baseline covariates between groups, followed by comparisons of two matched groups to meet the study aims. Finally, a competing risks model was established to estimate CIF. R software (version R-3.6.2) (Vienna, Austria) as well as SPSS, version 23.0 (SPSS Inc., Chicago, IL, USA), was employed for statistical analysis. GraphPad Prism 6.0 (GraphPad Software, San Diego, CA) was adopted to plot the survival curves. A two-sided *P <*0.05 indicated statistical significance.

## Results

### Baseline Features

Of the 10,092 eligible subjects receiving surgical resection due to T1 CRC, 5,208 patients were male, and the remaining 4,884 were female. The median age at diagnosis was 67 years, ranging from 18 to 101 years, and the mean ± SD of age was 66.31 ± 12.34 years. The median follow-up was 69 months, ranging between 2 and 155 months. The median number of sampled LNs was 13, ranging from 1 to 90. There were 3,669 patients (36.4%) with less than 12 examined lymph nodes (LNE <12) and 6,423 subjects (63.6%) with greater or equal to 12 examined lymph nodes (LNE ≥12). Patients 65 years or above were assigned into the elderly group. A total of 5,777 subjects (57.7%) were 65 years or older. Moreover, there were 3,546 subjects with greater or equal to 12 examined lymph nodes in the elderly patients. The observed rate of LNM was 14.9% (960 out of 6,423) in T1 CRC patients. The observed rate of LNM was 4.6% (15 out of 325) in T1 CRC elderly patients, with tumor size <3 cm, well differentiated, negative CEA level, and adenocarcinoma. The comparison of other clinicopathological characteristics of patients in the two groups showed a relevant imbalance (*P* < 0.001) (**Table 1**).

### Risk Factors of LNM

All patients underwent surgery, with at least 12 LNs sampled. To be specific, LNM risk was elevated in tumor size over 3 cm than tumor size under 1 cm (OR = 1.316, 95% CI: 1.016–1.706, *P* = 0.038). Patients with a negative CEA level had lower LNM risk than those with a positive CEA level (OR = 0.710, 95% CI: 0.553–0.911, *P* = 0.007). Moreover, elderly patients had a decreased LNM risk (age 65–79 years: OR = 0.633, 95% CI: 0.498–0.804; age over 80 years: OR = 0.477, 95% CI: 0.349–0.652, both *P <*0.001). Univariate and multivariate logistic regression models were employed for the identification of risk factors of LNM, revealing that age, histology, tumor site, CEA level, tumor size, and tumor grade were significant predictors for LNM. The detailed characteristics are displayed in **Table 2**. Furthermore, univariate and multivariate logistic regression models were employed for the identification of risk factors of LNM in elderly patients, showing that primary tumor site in the rectum/rectosigmoid had a higher LNM risk than that in the cecum (OR = 1.449, 95% CI: 1.043–2.013, *P* = 0.027). Tumor grade, histology, and CEA level were significant predictors for LNM. The detailed characteristics are displayed in **Table 3**.

**Table 2:** Logistic regression analysis of the risk factors for lymph node metastasis in T1 colorectal cancer (examined lymph nodes ≥12).

Characteristic	Univariate analysis		Multivariate analysis	
	OR (95% CI)	*P*	OR (95% CI)	*P*
Gender				
Female	Reference		Reference	
Male	0.999 (0.871–1.146)	0.988	0. 912 (0.790–1.053)	0.21
Age (years)				
Up to 49	Reference		Reference	
50–64	0.735 (0.586–0.923)	0.008	0.828 (0.655–1.047)	0.114
65–79	0.536 (0.426–0.674)	<0.001	0.633 (0.498–0.804)	<0.001
80+	0.394 (0.294–0.530)	<0.001	0.477 (0.349–0.652)	<0.001
Race				
White	Reference		Reference	
Black	1.132 (0.907–1.412)	0.272	1.194 (0.948–1.504)	0.132
Others^a^	1.434 (1.159–1.775)	0.001	1.305 (1.047–1.627)	0.018
Tumor size (cm)				
<1	Reference		Reference	
1–1.9	1.230 (0.966–1.566)	0.092	1.102 (0.859–1.413)	0.445
2–2.9	1.125 (0.876–1.446)	0.355	0.999 (0.771–1.295)	0.997
3+	1.469 (1.145–1.884)	0.002	1.316 (1.016–1.706)	0.038
Not stated	1.401 (1.065–1.843)	0.016	1.264 (0.946–1.687)	0.113
Year of diagnosis				
2004–2006	Reference		NI	
2007–2009	0.932 (0.758–1.146)	0.502		
2010–2012	0.959 (0.784–1.173)	0.685		
2013–2015	0.885 (0.724–1.082)	0.234		
Marital status				
Married	Reference		Reference	
Single/widowed	0.860 (0.729–1.015)	0.074	0.906 (0.760–1.081)	0.274
Other/unknown	0.789 (0.643–0.968)	0.023	0.769 (0.623–0.949)	0.014
Grade				
Well-differentiated	Reference		Reference	
Moderately differentiated	1.694 (1.354–2.120)	<0.001	1.638 (1.304–2.059)	<0.001
Poorly differentiated	3.838 (2.908–5.065)	<0.001	3.716 (2.786–4.957)	<0.001
Undifferentiated	2.507 (1.318–4.772)	0.005	2.341 (1.206–4.547)	0.012
Unknown	1.538 (1.077–2.196)	0.018	1.330 (0.915–1.932)	0.135
Primary site				
Cecum	Reference		Reference	
Ascending colon	0.719 (0.558–0.926)	0.011	0.751 (0.580–0.972)	0.030
Hepatic flexure	1.113 (0.742–1.670)	0.603	1.157 (0.764–1.750)	0.491
Transverse colon	0.725 (0.509–1.032)	0.074	0.751 (0.524–1.078)	0.121
Splenic flexure	1.356 (0.820–2.240)	0.235	1.345 (0.804–2.250)	0.259
Descending colon	0.973 (0.637–1.485)	0.899	0.910 (0.589–1.406)	0.671
Sigmoid colon	1.559 (1.248–1.946)	<0.001	1.496 (1.185–1.889)	0.001
Rectum/rectosigmoid junction	1.627 (1.303–2.033)	<0.001	1.504 (1.190–1.900)	0.001
CEA				
Positive	Reference		Reference	
Negative	0.756 (0.595–0.961)	0.022	0.710 (0.553–0.911)	0.007
Borderline/unknown	0.539 (0.423–0.687)	<0.001	0.547 (0.425–0.703)	<0.001
Histology				
Adenocarcinoma	Reference		Reference	
Mucinous carcinoma	1.496 (1.148–1.950)	0.003	1.695 (1.286–2.235)	<0.001
Signet ring cell carcinoma	3.163 (1.683–5.947)	<0.001	2.006 (1.017–3.957)	0.045

a American Indian/Alaska native, Asian/Pacific islander. NI, not included in the multivariate survival analysis.

**Table 3:** Logistic regression analysis of the risk factors for lymph node metastasis in T1 colorectal cancer in elderly patients (age ≥65 years; examined lymph nodes ≥12).

Characteristic	Univariate analysis		Multivariate analysis	
	OR (95% CI)	*P*	OR (95% CI)	*P*
Gender				
Female	Reference		Reference	
Male	1.061 (0.869–1.297)	0.560	1.025 (0.826–1.271)	0.823
Race				
White	Reference		Reference	
Black	1.262 (0.893–1.783)	0.187	1.415 (0.989–2.023)	0.057
Others^a^	1.435 (1.042–1.975)	0.027	1.334 (0.957–1.859)	0.089
Tumor size (cm)				
<1	Reference		Reference	
1–1.9	1.234 (0.859–1.772)	0.256	1.064 (0.734–1.541)	0.745
2–2.9	1.070 (0.733–1.561)	0.726	0.913 (0.619–1.347)	0.646
3+	1.569 (1.086–2.266)	0.016	1.320 (0.903–1.931)	0.152
Not stated	1.300 (0.847–1.995)	0.230	1.133 (0.722–1.777)	0.588
Year of diagnosis				
2004–2006	Reference		NI	
2007–2009	0.883 (0.659–1.183)	0.403		
2010–2012	0.856 (0.641–1.144)	0.294		
2013–2015	0.821 (0.617–1.092)	0.176		
Marital status				
Married	Reference		Reference	
Single/widowed	0.966 (0.771–1.210)	0.762	0.956 (0.751–1.217)	0.713
Other/unknown	0.814 (0.595–1.114)	0.199	0.804 (0.581–1.113)	0.189
Grade				
Well-differentiated	Reference		Reference	
Moderately differentiated	2.058 (1.417–2.989)	<0.001	1.997 (1.369–2.914)	<0.001
Poorly differentiated	5.737 (3.747–8.783)	<0.001	5.570 (3.607–8.600)	<0.001
Undifferentiated	5.045 (2.201–11.563)	<0.001	5.259 (2.257–12.253)	<0.001
Unknown	2.229 (1.272–3.905)	0.005	2.004 (1.116–3.596)	0.020
Primary site				
Cecum	Reference		Reference	
Ascending colon	0.784 (0.572–1.075)	0.131	0.826 (0.598–1.141)	0.246
Hepatic flexure	1.560 (0.984–2.473)	0.059	1.593 (0.992–2.556)	0.054
Transverse colon	0.753 (0.474–1.197)	0.230	0.792 (0.493–1.273)	0.336
Splenic flexure	1.345 (0.661–2.737)	0.414	1.313 (0.629–2.741)	0.468
Descending colon	0.830 (0.448–1.537)	0.553	0.879 (0.468–1.652)	0.690
Sigmoid colon	1.283 (0.934–1.762)	0.124	1.364 (0.982–1.895)	0.064
Rectum/rectosigmoid junction	1.418 (1.032–1.949)	0.031	1.449 (1.043–2.013)	0.027
CEA				
Positive	Reference		Reference	
Negative	0.824 (0.586–1.159)	0.266	0.828 (0.583–1.177)	0.294
Borderline/unknown	0.621 (0.442–0.873)	0.006	0.662 (0.465–0.942)	0.022
Histology				
Adenocarcinoma	Reference		Reference	
Mucinous carcinoma	1.418 (0.982–2.047)	0.062	1.484 (1.013–0.175)	0.043
Signet ring cell carcinoma	3.030 (1.249–7.352)	0.014	1.433 (0.551–3.726)	0.460

a American Indian/Alaska native, Asian/Pacific islander. NI, not included in the multivariate survival analysis.

### PSM for Elderly Patients

Adjustment of the observed effects in nonrandomized researches is critically involved in analyzing data in consideration of biased effect estimates due to confounding covariates. PSM was used to establish covariate balance and to minimize or even totally eliminate the confounding effects (11). After PSM, 1,733 of 2,231 patients in the LNE <12 group could be matched with 2,075 of the 3,546 in the LNE ≥12 group at a 1:2 ratio, suggesting that the relevant bias on the observed characteristics was lost in the two groups. Additionally, the baseline characteristics of the matched study population are displayed in **Table 4**.

**Table 4:** Baseline characteristics before and after the propensity score matching (1:2) of elderly patients (age ≥65 years).

Characteristic	Before matching		Statistic	*p*	After matching		Statistic	*p*
	Examined lymph nodes (LNE) <12	LNE ≥12			LNE <12	LNE ≥12		
	*N* = 2,231, %	*N* = 3,546, %			*N* = 1,733, %	*N* = 2,075, %		
Gender			*χ* ^2^ = 14.406	<0.001			*χ* ^2^ = 0.313	0.576
Female	1,076 (48.2)	1,892 (51.8)			871 (50.3)	1,024 (49.3)		
Male	1,155 (53.4)	1,654 (46.6)			862 (49.7)	1,051 (50.7)		
Race			*χ* ^2^ = 5.892	0.053			*χ* ^2^ = 0.012	0.994
White	1,800 (80.7)	2,936 (82.8)			1,399 (80.7)	1,677 (80.8)		
Black	224 (10.0)	292 (8.2)			166 (9.6)	199 (9.6)		
Others^a^	207 (9.3)	318 (9.0)			168 (9.7)	199 (9.6)		
LNM			*χ* ^2^ = 5.095	0.024			*χ* ^2^ = 4.326	0.038
No	1,999 (89.6)	3,108 (87.6)			1,509 (87.1)	1,852 (89.3)		
Yes	232 (10.4)	438 (12.4)			224 (12.9)	223 (10.7)		
Tumor size (cm)			*Z* = -0.190	0.849			*Z* = -1.151	0.250
<1	294 (13.2)	432 (12.2)			228 (13.2)	276 (13.3)		
1–1.9	704 (31.6)	1,051 (29.6)			496 (28.6)	650 (31.3)		
2–2.9	465 (20.8)	878 (24.8)			390 (22.5)	445 (21.4)		
3+	397 (17.8)	788 (22.2)			359 (20.7)	382 (18.4)		
Not stated	371 (16.6)	397 (11.2)			260 (15.0)	322 (15.5)		
Year of diagnosis			*Z* = -23.238	<0.001			*Z* = -4.002	<0.001
2004–2006	1,033 (46.3)	718 (20.2)			630 (36.5)	892 (43.0)		
2007–2009	570 (25.5)	873 (24.6)			490 (28.4)	556 (26.8)		
2010–2012	375 (16.8)	938 (26.5)			373 (21.6)	374 (18.0)		
2013–2015	253 (11.3)	1,017 (28.7)			234 (13.5)	253 (12.2)		
Marital status			*χ* ^2^ = 7.859	0.020			*χ* ^2^ = 1.799	0.407
Married	1,217 (54.5)	1,982 (55.9)			934 (53.9)	1,137 (54.8)		
Single/widowed	740 (33.2)	1,066 (30.1)			558 (32.2)	680 (32.8)		
Other/unknown	274 (12.3)	498 (14.0)			241 (13.9)	258 (12.4)		
Grade			*χ* ^2^ = 22.018	<0.001			*χ* ^2^ = 3.347	0.502
Well-differentiated	408 (18.3)	551 (15.5)			322 (18.4)	379 (18.3)		
Moderately differentiated	1,532 (68.7)	2,433 (68.6)			1,144 (67.3)	1,417 (68.3)		
Poorly differentiated	148 (6.6)	340 (9.6)			135 (7.3)	143 (6.9)		
Undifferentiated	17 (0.8)	37 (1.0)			17 (0.9)	17 (0.8)		
Unknown	126 (5.6)	185 (5.2)			115 (6.1)	119 (5.7)		
Primary site			*χ* ^2^ = 295.956	<0.001			*χ* ^2^ = 13.751	0.056
Cecum	295 (13.2)	708 (20.0)			289 (16.7)	290 (14.0)		
Ascending colon	301 (13.5)	954 (26.9)			282 (16.3)	300 (14.5)		
Hepatic flexure	61 (2.7)	169 (4.8)			61 (3.5)	61 (2.9)		
Transverse colon	223 (10.0)	286 (8.1)			177 (10.2)	204 (9.8)		
Splenic flexure	43 (1.9)	66 (1.9)			36 (2.1)	42 (2.0)		
Descending colon	134 (6.0)	131 (3.7)			92 (5.3)	121 (5.8)		
Sigmoid colon	664 (29.8)	639 (18.0)			430 (24.8)	592 (28.5)		
Rectum/rectosigmoid junction	510 (22.9)	593 (16.7)			366 (21.8)	465 (22.4)		
CEA			*χ* ^2^ = 16.617	<0.001			*χ* ^2^ = 1.204	0.548
Positive	177 (7.9)	304 (8.6)			158 (9.1)	170 (8.2)		
Negative	799 (35.8)	1,441 (40.6)			632 (36.5)	751 (36.2)		
Borderline/unknown	1,255 (56.3)	1,801 (50.8)			943 (54.4)	1,154 (55.6)		
Histology			*χ* ^2^ = 4.061	0.131			*χ* ^2^ = 4.173	0.124
Adenocarcinoma	2,102 (94.2)	3,293 (92.9)			1,604 (92.6)	1,953 (94.1)		
Mucinous carcinoma	117 (5.2)	229 (6.5)			113 (6.5)	110 (5.3)		
Signet ring cell carcinoma	12 (0.5)	24 (0.7)			16 (0.9)	12 (0.6)		

a American Indian/Alaska native, Asian/Pacific islander.

### Survival Analysis Before PSM in Elderly Patients

**Figure 1:**
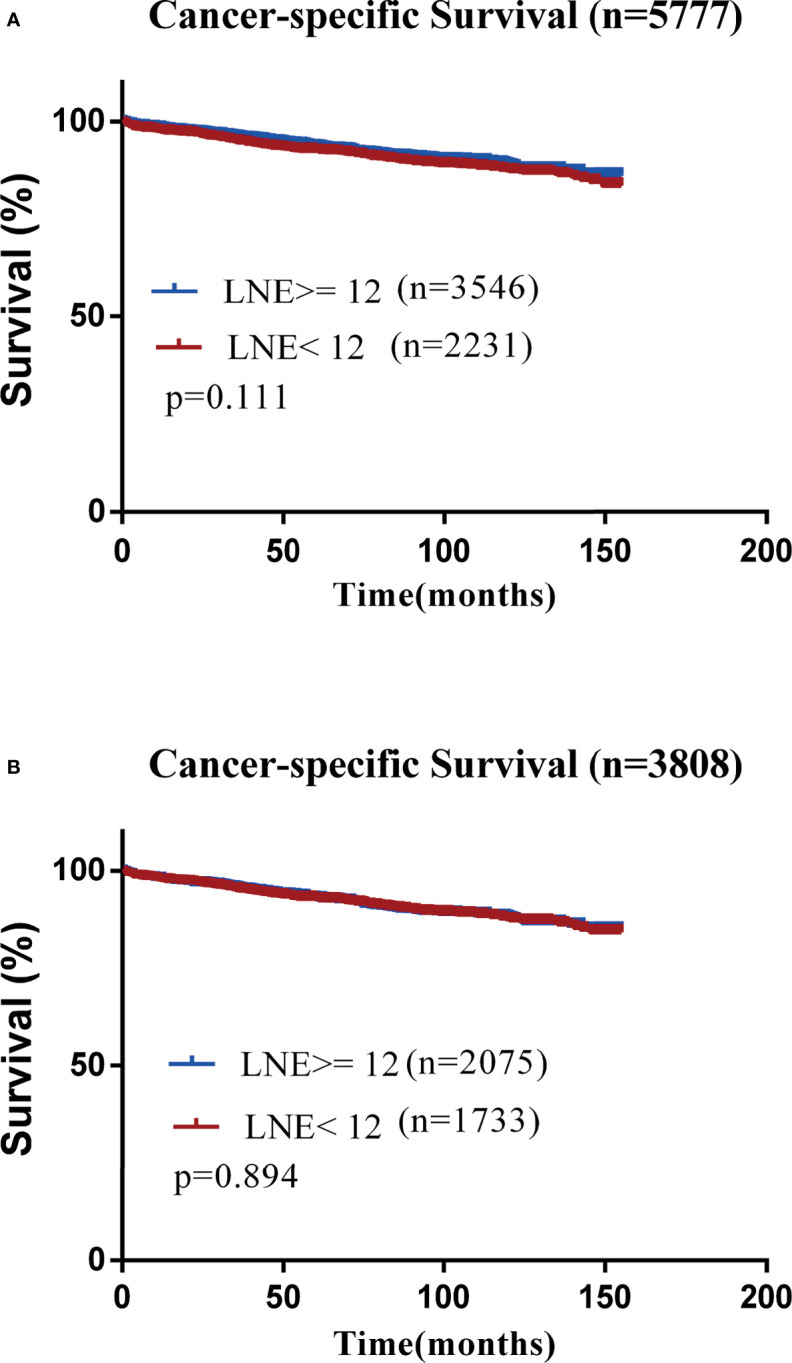
Kaplan–Meier curves for cancer-specific survival (CSS). **(A)** CSS in the original data set. **(B)** CSS after propensity score matching.

The mean CSS of elderly subjects receiving surgery with LNE ≥12 was insignificantly different from those with LNE <12 (142.91 months, 95% CI: 141.43–144.39 *versus* 141.13 months, 95% CI: 139.36–142.89, *P* = 0.11) (**Figure 1A**). In addition, a multivariate analysis on CSS of patients undergoing surgery with LNE ≥12 showed an insignificant survival benefit (HR = 0.865, 95% CI: 0.709–1.055, *P* = 0.153). Consistently, univariate and multivariate Cox regression analyses demonstrated that gender, tumor size, tumor grade, CEA level, LNM, and marital status were significant prognostic indicators for OS and CSS in elderly T1 CRC populations (**Table 5**).

**Table 5:** Cox regression analysis of prognostic factors for overall survival (OS) and cancer-specific survival (CSS) in T1 colorectal cancer of elderly patients (age ≥65 years).

Characteristic	OS				CSS			
	Univariate analysis		Multivariate analysis		Univariate analysis		Multivariate analysis	
	HR (95% CI)	*P*	HR (95% CI)	*P*	HR (95% CI)	*P*	HR (95% CI)	*P*
Gender								
Female	Reference		Reference		Reference		Reference	
Male	1.088 (0.997–1.188)	0.059	1.272 (1.159–1.397)	<0.001	1.133 (0.943–1.362)	0.182	1.245 (1.025–1.513)	0.027
Race								
White	Reference		Reference		Reference		Reference	
Black	1.057 (0.907–1.232)	0.480	0.962 (0.823–1.123)	0.621	1.501 (1.133–1.989)	0.005	1.476 (1.108–1.966)	0.008
Others^a^	0.719 (0.600–0.861)	<0.001	0.711 (0.593–0.852)	<0.001	0.932 (0.662–1.313)	0.689	0.860 (0.609–1.214)	0.392
Tumor size (cm)								
<1	Reference		Reference		Reference		Reference	
1–1.9	1.177 (1.002–1.383)	0.048	1.207 (1.026–1.420)	0.023	1.345 (0.935–1.933)	0.110	1.257 (0.872–1.810)	0.220
2–2.9	1.394 (1.182–1.643)	<0.001	1.418 (1.201–1.675)	<0.001	1.635 (1.132–2.363)	0.009	1.495 (1.031–2.167)	0.034
3+	1.531 (1.296–1.807)	<0.001	1.483 (1.253–1.755)	<0.001	2.280 (1.591–3.268)	<0.001	1.963 (1.363–2.828)	<0.001
Not stated	1.028 (0.853–1.238)	0.775	1.067 (0.883–1.290)	0.500	1.000 (0.649–1.540)	0.999	0.934 (0.602–1.449)	0.761
Year of diagnosis								
2004–2006	Reference		NI		Reference		Reference	
2007–2009	1.081 (0.972–1.202)	0.153			1.062 (0.851–1.324)	0.594	1.109 (0.885–1.390)	0.368
2010–2012	1.014 (0.887–1.160)	0.837			0.820 (0.625–1.077)	0.154	0.877 (0.662–1.162)	0.362
2013–2015	0.829 (0.679–1.012)	0.065			0.624 (0.424–0.918)	0.017	0.683 (0.460–1.014)	0.058
Marital status								
Married	Reference		Reference		Reference		Reference	
Single/widowed	1.575 (1.433–1.730)	<0.001	1.646 (1.490–1.819)	<0.001	1.448 (1.187–1.766)	<0.001	1.454 (1.177–1.796)	0.001
Other/unknown	1.073 (0.928–1.240)	0.341	1.109 (0.958–1.285)	0.166	1.111 (0.829–1.488)	0.482	1.076 (0.798–1.451)	0.631
Lymph node metastases								
No	Reference		Reference		Reference		Reference	
Yes	1.045 (0.914–1.195)	0.521	1.063 (0.928–1.218)	0.380	1.937 (1.542–2.433)	<0.001	1.755 (1.390–2.215)	<0.001
Number of examined lymph nodes								
LNE<12	Reference				Reference		Reference	
LNE≥12	0.870 (0.796–0.951)	.002	0.834 (0.758–0.917)	<0.001	0.860 (0.715–1.035)	0.111	0.865 (0.709–1.055)	0.153
Grade								
Well-differentiated	Reference		Reference		Reference		Reference	
Moderately differentiated	1.042 (0.924–1.173)	0.504	1.050 (0.930–1.185)	0.431	1.221 (0.931–1.601)	0.149	1.130 (0.858–1.489)	0.384
Poorly differentiated	1.010 (0.841–1.213)	0.915	1.050 (0.930–1.185)	0.720	1.939 (1.366–2.754)	<0.001	1.746 (1.216–2.508)	0.003
Undifferentiated	1.220 (0.749–1.986)	0.425	1.235 (0.756–2.018)	0.400	1.779 (0.715–4.424)	0.215	1.713 (0.685–4.287)	0.250
Unknown	1.220 (0.749–1.986)	0.050	0.849 (0.668–1.078)	0.180	0.852 (0.505–1.439)	0.550	0.961 (0.560–1.648)	0.884
Primary site								
Cecum	Reference		Reference		Reference		Reference	
Ascending colon	0.916 (0.797–1.054)	0.220	0.955 (0.830–1.099)	0.518	0.996 (0.727–1.364)	0.979	1.095 (0.798–1.503)	0.574
Hepatic flexure	0.944 (0.746–1.194)	0.629	0.966 (0.763–1.223)	0.775	0.944 (0.548–1.625)	0.835	0.985 (0.571–1.699)	0.957
Transverse colon	0.899 (0.749–1.079)	0.255	0.918 (0.764–1.104)	0.365	0.789 (0.509–1.223)	0.290	0.904 (0.581–1.408)	0.655
Splenic flexure	1.115 (0.815–1.524)	0.496	1.199 (0.876–1.642)	0.257	1.066 (0.513–2.215)	0.864	1.161 (0.557–2.423)	0.690
Descending colon	0.798 (0.627–1.017)	0.068	0.836 (0.655–1.067)	0.150	0.964 (0.574–1.618)	0.888	1.098 (0.651–1.855)	0.725
Sigmoid colon	0.794 (0.691–0.913)	0.001	0.794 (0.688–0.917)	0.002	1.035 (0.763–1.403)	0.825	1.093 (0.799–1.495)	0.579
Rectum/rectosigmoid junction	0.882 (0.766–1.017)	0.084	0.853 (0.738–0.986)	0.032	1.515 (1.131–2.029)	0.005	1.487 (1.102–2.007)	0.009
Carcinoembryonic antigen								
Positive	Reference		Reference		Reference		Reference	
Negative	0.592 (0.508–0.689)	<0.001	0.616 (0.528–0.719)	<0.001	0.543 (0.404–0.731)	<0.001	0.583 (0.432–0.786)	<0.001
Borderline/unknown	0.650 (0.561–0.753)	<0.001	0.684 (0.589–0.793)	<0.001	0.517 (0.388–0.688)	<0.001	0.597 (0.446–0.799)	0.001
Histology								
Adenocarcinoma	Reference		Reference		Reference		Reference	
Mucinous carcinoma	1.149 (0.966–1.366)	0.117	1.170 (0.981–1.395)	0.080	1.140 (0.792–1.642)	0.480	1.200 (0.827–1.740)	0.337
Signet ring cell carcinoma	0.781 (0.432–1.413)	0.415	0.799 (0.437–1.461)	0.466	1.286 (0.481–3.444)	0.616	0.788 (0.287–2.165)	0.644

a American Indian/Alaska native, Asian/Pacific islander. NI, not included in the multivariate survival analysis.

### Survival Analysis After PSM in Elderly Patients

In this cohort, the mean OS of elderly patients receiving surgery with LNE ≥12 was insignificantly different from those with LNE <12 (107.79 months, 95% CI: 105.06–110.52 *versus* 104.47 months, 95% CI: 101.95–106.98, *P* = 0.118). The mean CSS of subjects undergoing surgery with LNE <12 was insignificantly different from those with LNE ≥12 (141.24 months, 95% CI: 139.41–143.07 *versus* 141.44 months, 95% CI: 139.43–143.44, *P* = 0.894) (**Figure 1B**). The multivariate analysis revealed no significantly different OS or CSS between elderly patients receiving surgery with LNE ≥12 and those with LNE <12 (OS: HR = 0.904, 95% CI: 0.816–1.001, *P* = 0.052; CSS: HR = 0.955, 95% CI: 0.772–1.181, *P* = 0.668). The characteristics are displayed in **Table 6** in detail.

**Table 6:** Cox regression analysis of prognostic factors for overall survival (OS) and cancer-specific survival (CSS) in T1 colorectal cancer of elderly patients (age ≥65 years) after propensity score matching.

Characteristic	OS				CSS			
	Univariate analysis		Multivariate analysis		Univariate analysis		Multivariate analysis	
	HR (95% CI)	*P*	HR (95% CI)	*P*	HR (95% CI)	*P*	HR (95% CI)	*P*
Gender								
Female	Reference		Reference		Reference		Reference	
Male	1.095 (0.991–1.211)	0.075	1.302 (1.171–1.448)	<0.001	1.104 (0.895–1.362)	0.355	1.258 (1.007–1.572)	0.043
Race								
White	Reference		Reference		Reference		Reference	
Black	1.032 (0.869–1.227)	0.718	0.933 (0.783–1.112)	0.436	1.561 (1.146–2.127)	0.005	1.549 (1.129–2.124)	0.007
Others^a^	0.685 (0.559–0.838)	<0.001	0.687 (0.560–0.842)	<0.001	0.880 (0.596–1.298)	0.518	0.830 (0.561–1.228)	0.351
Tumor size (cm)								
<1	Reference		Reference		Reference		Reference	
1–1.9	1.146 (0.958–1.371)	0.137	1.164 (0.971–1.394)	0.100	1.420 (0.944–2.136)	0.092	1.353 (0.898–2.039)	0.148
2–2.9	1.331 (1.107–1.601)	0.002	1.344 (1.115–1.620)	0.002	1.610 (1.059–2.448)	0.026	1.461 (0.957–2.230)	0.079
3+	1.415 (1.174–1.705)	<0.001	1.318 (1.091–1.593)	0.004	2.383 (1.588–3.575)	<0.001	2.008 (1.331–3.030)	0.001
Not stated	1.020 (0.832–1.250)	0.851	1.090 (0.885–1.342)	0.419	0.915 (0.560–1.495)	0.722	0.900 (0.546–1.484)	0.679
Year of diagnosis								
2004–2006	Reference		NI		Reference		NI	
2007–2009	1.081 (0.959–1.218)	0.201			1.083 (0.846–1.386)	0.527		
2010–2012	1.136 (0.968–1.334)	0.119			0.950 (0.687–1.313)	0.754		
2013–2015	1.050 (0.799–1.379)	0.726			0.904 (0.547–1.494)	0.694		
Marital status								
Married	Reference		Reference		Reference		Reference	
Single/widowed	1.608 (1.444–1.790)	<0.001	1.705 (1.521–1.913)	<0.001	1.541 (1.230–1.931)	<0.001	1.532 (1.203–1.950)	0.001
Other/unknown	1.166 (0.992–1.370)	0.063	1.232 (1.045–1.452)	0.013	1.219 (0.877–1.694)	0.239	1.165 (0.831–1.633)	0.375
Lymph node metastases								
No	Reference		Reference		Reference		Reference	
Yes	1.013 (0.869–1.181)	0.864	1.038 (0.888–1.214)	0.637	1.660 (1.266–2.176)	<0.001	1.544 1.171 2.037	0.002
Number of examined lymph nodes								
Examined lymph nodes (LNE) <12	Reference		Reference		Reference		Reference	
LNE ≥12	0.923 (0.834–1.021)	0.119	0.904 (0.816–1.001)	0.052	0.986 (0.798–1.217)	0.894	0.955 (0.772–1.181)	0.668
Grade								
Well-differentiated	Reference		Reference		Reference		Reference	
Moderately differentiated	1.138 (0.994–1.302)	.061	1.140 (0.994–1.308)	0.061	1.332 .985 1.802	0.062	1.212 (0.891–1.647)	0.221
Poorly differentiated	1.082 (0.872–1.342)	0.474	1.078 (0.864–1.345)	0.505	1.790 (1.176–2.724)	0.007	1.526 (0.987–2.360)	0.057
Undifferentiated	1.168 (0.655–2.083)	0.599	1.221 (0.680–2.193)	0.504	0.951 (0.231–3.906)	0.944	0.916 (0.221–3.797)	0.904
Unknown	0.790 (0.609–1.023)	0.074	0.822 (0.628–1.077)	0.155	0.85 (0.479–1.514)	0.584	0.958 (0.528–1.738)	0.888
Primary site								
Cecum	Reference		Reference		Reference		Reference	
Ascending colon	0.842 (0.712–0.997)	0.046	0.868 (0.732–.028)	0.101	0.823 (0.561–1.207)	0.318	0.887 (0.603–1.306)	0.543
Hepatic flexure	0.850 (0.638–1.132)	0.265	0.837 (0.627–1.116)	0.224	0.867 (0.454–1.656)	0.666	0.889 (0.464–1.700)	0.721
Transverse colon	0.791 (0.646–0.969)	0.023	0.814 (0.663–0.999)	0.049	0.620 (0.378–1.015)	0.057	0.720 (0.437–1.184)	0.196
Splenic flexure	0.994 (0.709–1.395)	0.972	1.097 (0.781–1.542)	0.594	0.794 (0.342–1.843)	0.591	0.878 (0.377–2.047)	0.763
Descending colon	0.717 (0.552–0.932)	0.013	0.767 (0.590–0.999)	0.049	0.769 (0.435–1.359)	0.366	0.891 (0.502–1.584)	0.695
Sigmoid colon	0.680 (0.582–0.795)	<0.001	0.693 (0.591–0.812)	<0.001	0.836 (0.597–1.171)	0.298	0.915 (0.649–1.291)	0.614
Rectum/Rectosigmoid junction	0.766 (0.654–0.898)	0.001	0.747 (0.636–0.878)	<0.001	0.836 (0.597–1.171)	0.181	1.248 (0.896–1.739)	0.190
Carcinoembryonic antigen								
Positive	Reference		Reference		Reference		Reference	
Negative	0.617 (0.518–0.735)	<0.001	0.635 (0.532–0.757)	<0.001	0.536 (0.382–0.752)	<0.001	0.585 (0.415–0.825)	0.002
Borderline/unknown	0.652 (0.551–0.771)	<0.001	0.670 (0.566–0.794)	<0.001	0.526 (0.380–0.727)	<0.001	0.618 (0.444–0.859)	0.004
Histology								
Adenocarcinoma	Reference		Reference		Reference		Reference	
Mucinous carcinoma	1.141 (0.936–1.391)	0.193	1.167 (0.954–1.428)	0.133	1.105 (0.724–1.686)	0.644	1.148 (0.746–1.768)	0.530
Signet ring cell carcinoma	0.795 (0.427–1.481)	0.471	0.863 (0.457–1.631)	0.650	1.425 (0.531–3.819)	0.482	1.206 (0.434–3.354)	0.719

a American Indian/Alaska native, Asian/Pacific islander. NI, not included in the multivariate survival analysis.

### Competing Risks Analysis After PSM in Elderly Patients

**Figure 2:**
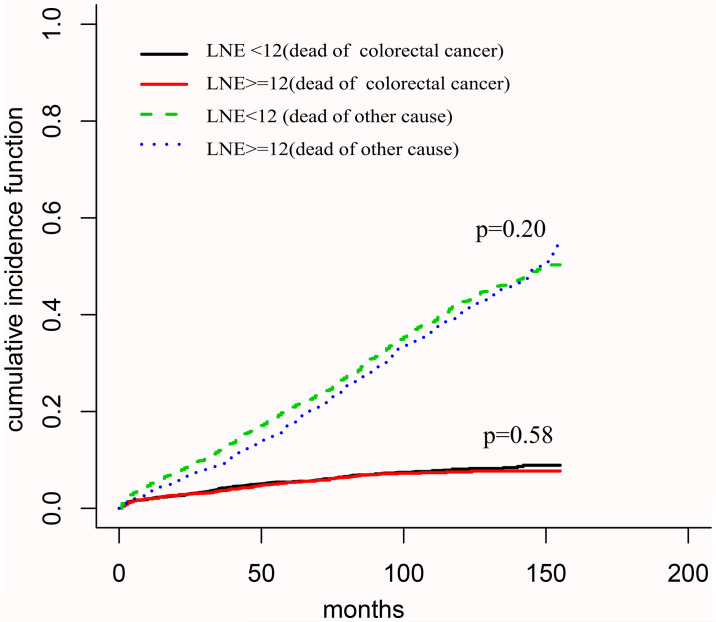
Cumulative incidence function for death in elderly patients with T1 colorectal cancer after surgery.

Both oncological and non-oncological factors could affect the survival outcomes of tumor patients. In other words, tumor patients may die from a non-oncological cause (12). To this end, a competing risks model was adopted to precisely assess the prognostic value of LNE on elderly T1 CRC patients, which could directly connect the impacts of risk factors with cause-specific cumulative incidence of mortality (13). Consequently, the survival in the LNE ≥12 group was no longer than that in LNE <12 group (subdistribution hazard ratio, SHR = 0.891, 95% CI: 0.693–1.145, *P* = 0.37). Normal CEA level (SHR = 0.568, 95% CI: 0.385–0.837, *P* = 0.0043), tumor size >3.0 cm (SHR = 2.289, 95% CI: 1.388–3.776, *P* = 0.026), poor differentiation (SHR = 1.664, 95% CI: 1.013–2.733, *P* = 0.044), and primary tumor site in the rectum (SHR = 1.772, 95% CI: 1.204–2.607, *P* = 0.0037) were significant prognostic indicators for elderly T1 CRC patients. Other detailed characteristics are shown in **Table 7**. CIF was additionally employed for assessing the possibility of death caused by oncological and non-oncological events (14). Consequently, the oncological and non-oncological death rates were insignificantly different between patients with LNE ≥12 and those with LNE <12 (**Figure 2**).

**Table 7:** Competing risks analysis for cancer-specific death in T1 colorectal cancer of elderly patients (age ≥65 years) after propensity score matching.

Characteristic	Multivariate analysis	
	SHR (95% CI)	*P*
Gender		
Female	Reference	
Male	1.131 (0.878–1.457)	0.34
Race		
White	Reference	
Black	1.808 (1.262–2.590)	0.0012
Others^a^	0.932 (0.599–1.449)	0.75
Tumor size (cm)		
<1	Reference	
1–1.9	1.269 (0.778–2.071)	0.34
2–2.9	1.584 (0.957–2.623)	0.074
3+	2.289 (1.388–3.776)	0.0012
Not stated	0.663 (0.352–1.248)	0.2
Year of diagnosis		
2004–2006	Reference	
2007–2009	1.094 (0.821–1.456)	0.95
2010–2012	0.988 (0.685–1.425)	0.37
2013–2015	0.771 (0.437–1.361)	0.75
Marital status		
Married	Reference	
Single/widowed	1.248 (0.949–1.640)	0.11
Other/unknown	1.032 (0.696–1.529)	0.88
Lymph node metastasis		
No	Reference	
Yes	1.857 (1.374–2.509)	<0.001
LNE		
LNE<12	Reference	
LNE≥12	0.891 (0.693–1.145)	0.37
Grade		
Well-differentiated	Reference	
Moderately differentiated	1.175 (0.816–1.693)	0.39
Poorly differentiated	1.664 (1.013–2.733)	0.044
Undifferentiated	0.612 (0.079–4.743)	0.64
Unknown	1.244 (0.640–2.416)	0.52
Primary site		
Cecum	Reference	
Ascending colon	0.924 (0.567–1.506)	0.75
Hepatic flexure	0.784 (0.325–1.889)	0.59
Transverse colon	0.753 (0.404–1.404)	0.37
Splenic flexure	1.101 (0.423–2.864)	0.84
Descending colon	1.175 (0.602–2.294)	0.64
Sigmoid colon	1.192 (0.789–1.802)	0.40
Rectum/Rectosigmoid junction	1.772 (1.204–2.607)	0.0037
CEA		
Positive	Reference	
Negative	0.568 (0.385–0.837)	0.0043
Borderline/unknown	0.612 (0.421–0.889)	0.0099
Histology		
Adenocarcinoma	Reference	
Mucinous carcinoma	1.097 (0.658–1.828)	0.72
Signet ring cell carcinoma	1.101 (0.372–3.255)	0.86

LNE, number of examined lymph nodes; SHR, subdistribution hazard ratio; 95% CI, 95% confidence intervals; CEA, carcinoembryonic antigen.

a American Indian/Alaska native, Asian/Pacific islander.

## Discussion

Surgical resection and endoscopic submucosal dissection are the main therapeutic approaches for T1 CRC. Despite LN dissection during surgical intervention, 2.3–4% of T1 CRC patients still suffer from post-operative relapse (15). An endoscopic resection of early-stage CRC including mucosal and submucosal cancer is advantageous, which could dramatically decrease postoperative morbidity, improve life quality, and provide almost comparable long-term clinical outcomes compared with radical surgery (6, 16). Notably, great caution should be given to endoscopic resection indications in T1 CRC in consideration of LNM in nearly one-tenth of T1 CRC patients (17). According to the Japanese Society for Cancer of the Colon and Rectum guideline, the presence of any of the four factors (lymphovascular invasion, budding, tumor invasion depth as well as poor histology) (18) indicates the recommendation of additional surgery for LN dissection. Except for its effect on prognosis, the benefit of surgical resection is limited, particularly for patients of advanced age or with severe comorbidities.

The risk factors for LNM were identified by logistic regression analysis. Patients with an inadequate number of sampled LN were eliminated during the selection process (the cutoff value was set at 12 on the basis that at least 12 LN exams are generally required for precise pathological diagnosis) (3). In our study, the LNM rate was 14.9% (960 out of 6,423), which was remarkably higher than previously reported in T1 CRC patients (about 10%) (17). The inconsistency might be caused by our present inclusion criteria, that is, only patients receiving radical surgery were enrolled in our research. To further attenuate the risk of false-negative LNM and downgrading after neoadjuvant chemoradiation, patients with inadequate LNs sampled and those undergoing preoperative radiotherapy were eliminated from our research, which could give rise to more reliable LNM rates than the previous ones.

In the present population-based research, we comprehensively examined the predictors of regional LNM in T1 CRC patients undergoing surgery and having at least 12 LNs sampled. Histology, tumor grade, tumor size, CEA, race, primary tumor location, and age were significant predictors for LNM. MAC and SRCC are relatively rare pathological types of CRC, accounting for about 10–15 and 0.1–2.4% of all CRC cases, respectively (19). As a distinct subtype, MAC and SRCC have been showed to be associated with higher risks of lymph node involvement in stage I and II colorectal cancer (20). Here we consistently showed a higher LNM risk in patients with colorectal MAC and SRCC (OR = 1.695, 95% CI: 1.286–2.235, *P* < 0.001 and OR = 2.006, 95% CI: 1.017–3.957, *P* = 0.045). In addition, the LNM risk was significantly lower in well-differentiated tumors than in moderately or poorly differentiated or undifferentiated tumors, which was consistent with previous findings in T1 rectal and colon cancer (21). Furthermore, only tumor size ≥3 cm showed an elevated risk of regional LNM (OR = 1.316, 95% CI: 1.016–1.706, *P* = 0.038). Like other studies concerning colorectal cancer (21–23), our discovery that tumor size was a predictive factor for the risk of LNM in T1 colorectal cancer was consistent.

The primary tumor site has long been demonstrated to influence LNM risk in CRC. However, the prognostic significance and LNM relevance of laterality in T1 CRC (mainly including the left hemi-colon, right hemi-colon, and rectum) have been explored, giving rise to controversial outcomes (24). Therefore, the whole colorectal tract was divided into eight sections to determine the possible correlation between tumor sites and diverse clinical variables. As a result, elderly patients with rectum/rectosigmoid junction cancer had a higher LNM risk than those with cecum cancer. The LNM risk in T1 rectal carcinoma has been shown to be as high as 15% (25, 26), declining to 8% in the left colon and 3% in the right colon (25). Here we report alike consequences, which suggests that carcinoma of the ascending colon significantly decreases the LNM risk, whereas rectum/rectosigmoid junction cancer significantly increases the LNM risk.

Consistent with previous results in T1 CRC (21), we also found older age as a significant negative predictor for LNM. To be specific, the LNM risk of patients 65–79 years old and over 80 years declined to 0.63 and 0.47, respectively, in comparison to those under 49 years (both *P* < 0.001). The survival of CRC patients is affected by diverse prognostic factors. Surgical resection, a major therapy for CRC, might be improper or unsafe for elderly patients with comorbidities. Endoscopic resection has instead been proposed as a minimally invasive technique for precancerous lesions as well as early-stage CRC. The LNM rate correlates with the infiltration depth of the submucosa (Sm). While Sm1 tumors have a LNM rate of 3.4%, the rate goes up to 22.6% if the lower third of the Sm is infiltrated (27), while CRC Sm1 tumors have a LNM rate of 3.4%, similar to our findings of tumor size <3 cm, well differentiated, negative CEA level, and adenocarcinoma in elderly patients with T1 colorectal cancer of LNM rate 4.6% (15 out of 325).

The survival analysis revealed that LNM was a significant prognostic indicator for CSS (HR = 1.755, 95% CI: 1.390–2.215, *P* < 0.001) but not for OS (HR = 1.063, 95% CI: 0.928–1.218, *P* = 0.380) in elderly patients. Meanwhile, LNE ≥12 was a significant positive indicator for OS in comparison with LNE <12 (HR = 0.834, 95% CI: 0.758–0.917, *P* < 0.001) but not for CSS. Nevertheless, after PSM adjustment, OS (HR = 0.904, 95% CI: 0.816–1.001, *P* = 0.052) or CSS (HR = 0.955, 95% CI: 0.772–1.181, *P* = 0.668) was not significantly different between LNE ≥12 and LNE <12. Moreover, the univariate and multivariate Cox regression analyses also revealed tumor size, CEA level, race as well as marital status as significant indicators for OS and CSS.

In elderly tumor patients, various factors could cause the existence of right censoring, including loss of follow-up and no death, which do not prevent the survival or death of patients. By contrast, when patients die from non-oncological causes during follow-up, the proportion of cause-specific death (CSD) is decreased. The application of right censored data using conventional regression survival analysis can lead to biases, generally causing an overestimation of the possibility of CSD. Unfortunately, the abovementioned concern is frequently observed in prognostic prediction among the elderly, who are more vulnerable to frailty and comorbidities and have elevated non-oncological death than the other age groups. Under this situation, the competing risks concept might be used to readily solve the problem (28). For multivariate analysis, the two most commonly applied approaches include cause-specific hazard function and proportional subdistribution hazard function. The latter renders the covariant effects as a better and more intuitive explanation, which can be properly used to calculate the risk score and to construct a clinical prediction model (29). In terms of predictive factors, LNE ≥12 was not significantly better than LNE <12 (SHR = 0.891, 95% CI: 0.693–1.145, *P* = 0.37). Consistently with previous outcomes, we also find a negative correlation between tumor size ≥3 cm and survival (SHR = 2.289, 95% CI: 1.388–3.776, *P* = 0.026), which is suggestive that tumor size could reflect tumor invasiveness to a certain degree (30). Furthermore, our study showed that primary tumor site in the rectum (SHR = 1.772, 95% CI: 1.204–2.607, *P* = 0.0037) was significantly worse than in the cecum for elderly T1 CRC patients. It corresponded with the LNM risk in T1 rectal carcinoma, higher than in the left colon or in right colon (25, 26). Preoperative CEA and histology have been prevalently accepted as independent prognostic indicators for CRC, capable of an effective prognostic prediction in CRC. A positive CEA level and poorly differentiated histology are independent influencing factors for CRC prognosis. The prognostic value of these variables is also reflected in our model.

In this population-based research, our findings are mainly based on real-world outcomes. To our knowledge, this is the first population-based study to describe the influence of old age on risk of lymph node metastasis and survival in patients with T1 colorectal cancer. Nevertheless, certain limitations must be acknowledged. Firstly, relevant data on lymphovascular invasion, the deep distance of submucosal invasion, and macroscopic type are inaccessible in the SEER database, which are potential risk factors for LNM. The absence of these variables might affect the accurate assessment of LNM. Secondly, the applied models are simplified and only use available and accepted measures, which clearly do not adequately account for all variables associated with subject outcomes. Of course, we excluded patients who died within 1 month after surgery to reduce the impact of surgical complications. Additionally, older patients and those with a higher comorbidity index had lower odds of being treated with surgery. Selection biases are unavoidable in the retrospective analysis. Therefore, we applied the PSM, and competing risks analysis was used to analyze the associations between old age and lymph node status and to validate the prognostic value of old age on cancer-specific survival to reduce bias as much as possible. Finally, although PSM was further performed in this study, the results must be cautiously interpreted due to the fraction of unmatched patients.

## Conclusion

In summary, in this population-based analysis on T1 CRC patients after surgery, the decreased morbidity for local excision has to be weighed against the favorable outcomes. The predictive value of tumor size, grading, primary site, histology, CEA level, and age for LNM should be considered in medical decision making about local resection. Tumor size <3 cm, well differentiated, negative CEA level, and adenocarcinoma could be used to properly select elderly colorectal cancer patients for local excision.

## Data Availability Statement

The original contributions presented in the study are included in the article/supplementary material. Further inquiries can be directed to the corresponding author.

## Ethics Statement

Access to SEER database was obtained, and our study gained institutional approval. The patients/participants provided their written informed consent to participate in this study. Written informed consent was obtained from the individual(s) for the publication of any potentially identifiable images or data included in this article.

## Author Contributions

HY, PC, and QZ participated in the design of this project, interpretation of data, and drafting and critical revision of the article and provided final approval of the version to be submitted. HY and BZ completed the data collection and analysis. All authors contributed to the article and approved the submitted version.

## Conflict of Interest

The authors declare that the research was conducted in the absence of any commercial or financial relationships that could be construed as a potential conflict of interest.

## Publisher’s Note

All claims expressed in this article are solely those of the authors and do not necessarily represent those of their affiliated organizations, or those of the publisher, the editors and the reviewers. Any product that may be evaluated in this article, or claim that may be made by its manufacturer, is not guaranteed or endorsed by the publisher.
